# Draft Genome Sequences of *Propionibacterium acnes* Type Strain ATCC6919 and Antibiotic-Resistant Strain HL411PA1

**DOI:** 10.1128/genomeA.00740-14

**Published:** 2014-08-14

**Authors:** Jared Liu, Alison Cheng, Nathanael Joshua Bangayan, Emma Barnard, Emily Curd, Noah Craft, Huiying Li

**Affiliations:** a Department of Molecular and Medical Pharmacology, Crump Institute for Molecular Imaging, David Geffen School of Medicine, University of California Los Angeles, Los Angeles, California, USA; b Department of Ecology and Evolutionary Biology, University of California Los Angeles, Los Angeles, California, USA; c Los Angeles Biomedical Research Institute at Harbor-UCLA Medical Center, Torrance, California, USA; d UCLA-DOE Institute for Genomics and Proteomics, Los Angeles, California, USA

## Abstract

*Propionibacterium acnes* is a major skin commensal and is associated with acne vulgaris, the most common skin disease. Here we report the draft genome sequences of two *P. acnes* strains, the type strain ATCC6919 and an antibiotic-resistant strain, HL411PA1.

## GENOME ANNOUNCEMENT

*Propionibacterium acnes* is a bacterial species commonly found on human skin. It has been linked to acne pathogenesis. The *P. acnes* type strain ATCC6919 (also known as NCTC737 and DSM1897) was isolated by A. F. Hayden from facial acne (1, 2) and has been a widely used lab strain since the early characterization of *P. acnes* in the 1960s (2–4). As no genomic information was available for ATCC6919, we sequenced this type strain to better understand its documented properties and its virulence potential.

Antibiotic-resistant *P. acnes* strains have been emerging since the 1970s after antibiotics were introduced as one of the main treatments for acne. We isolated a *P. acnes* strain, HL411PA1, from the nose skin of an acne patient who reported no previous antibiotic treatment for acne. However, HL411PA1 is resistant to the major antibiotics used in acne treatment, including tetracycline (MIC 48 µg/mL), erythromycin (MIC >256 µg/mL), and clindamycin (MIC >256 µg/mL), but not minocycline (MIC 0.5 µg/mL). This finding supports previous evidence that antibiotic-resistant strains can be transmitted between individuals (5, 6). We sequenced the genome of HL411PA1 to determine whether this antibiotic-resistant strain encodes novel virulence factors.

The genomes of ATCC6919 and HL411PA1 were sequenced using Illumina MiSeq. The sequence reads were pair-ended with 250 nucleotides per read. Both genomes were assembled using MIRA v3.2.1 (7) with manual inspections using Consed (8). Gene annotation was performed using GeneMark HMM (9) and Glimmer 3.02 (10). Transfer RNAs (tRNAs) and transfer messenger RNAs (tmRNAs) were predicted using Aragorn (11). The ATCC6919 genome is 2,602,215 bp long (260 contigs, *N*_50_ of 50,832) with 60.1% G+C content and 2,620 predicted open reading frames (ORFs). The sequencing coverage is 72×. The HL411PA1 genome is 2,497,951 bp long (55 contigs, *N*_50_ of 174,713) with 60.0% G+C content and 2,369 predicted ORFs. The sequencing coverage is 118×.

The genomes of ATCC6919 and HL411PA1 are highly similar to previously sequenced *P. acnes* genomes (6). ATCC6919 and HL411PA1 both cluster with strains from clade IA-1 (12). Based on the 16S rRNA sequences, ATCC6919 belongs to ribotype (RT) 1, a strain type that is prevalent in both acne patients and healthy individuals (12). HL411PA1 belongs to RT5, a strain type associated with acne (12). However, the genome of HL411PA1 is atypical for RT5 in that it lacks any of the three loci found in RT5 strains (6, 12). These loci likely originated from mobile genetic elements and encode putative virulence genes (13).

As ATCC6919 has been widely used in *P. acnes*-related studies, its sequence will provide useful genomic context and insight for both its documented and yet to be discovered properties. The genome of HL411PA1 may contribute to future investigations of antibiotic-resistant *P. acnes* strains in disease pathogenesis.

### Nucleotide sequence accession numbers.

The whole-genome shotgun projects for ATCC6919 and HL411PA1 were deposited at DDBJ/EMBL/GenBank under the accession numbers JNHS00000000 and JNHT00000000, respectively.
